# Supplementary material for: Histone gene replacement reveals a post-transcriptional role for H3K36 in maintaining metazoan transcriptome fidelity
Source: eLife. 2017 Mar 27;6:e23249. doi: 10.7554/eLife.23249 (PMC5404926; doi:10.7554/eLife.23249)
Supplement: Supplementary file 2. — DOI: http://dx.doi.org/10.7554/eLife.23249.017 [file elife-23249-supp2.docx]

**Table 2: List of primer sequences**

CG17654_F: CATCTTCATGGCCTCTGTGA

CG17654_R: TGCCGCTGTACAAACACATT

CG2229_F: TCTGGGACATGATCTGGACA

CG2229_R: CCTGGTCAACAAGGTGGAGT

CG5765_F: AGTCTCGCCTCCCTTTAAGC

CG5765_R: TTGATGGCCTGGAATTCTTC

CG34278_F: CTTCTGCGGCATTGTAGGAT

CG34278_R: GACTTGTCCACAGGGTCCTT

CG11129_F: ACATGGGTCTCCAGATCGAC

CG11129_R: CTACGCATTCCGGGTTTTTA

CG32073_F: ACGGTGGTGGTTGTGGTAGT

CG32073_R: GTGGCCATTTGCCTGTATTC

YFP_232F: CACATGAAGCAGCACGACTTC

YFP_688R: GCTCGTCCATGCCGAGAGTGAT

Act5C-87E_F: GGCACCACACCTTCTACAATGAGC

Act5C-87E_R: GAGGCGTACAGCGAGAGCACAG

lmPAT_phospho-anchor: pCAGCUGUAGCUAUGCGCACCGAGUCAGAUCAG

lmPAT_RTanchor: CTGATCTGACTCGGTGCGCA

lmPAT_RTnested: TGCGCATAGCTACAGCTGTTTT

lmPAT_TVN: GCGAGCTCCGCGGCCGCGTTTTTTTTTTTTVN

YFP_lmPAT_2786F: TGCTCAGAAGAAATGCCATC

YFP_lmPAT_3098F: CCCCCTGAACCTGAAACATA
